# Clinical impact of depression and anxiety in patients with idiopathic pulmonary fibrosis

**DOI:** 10.1371/journal.pone.0184300

**Published:** 2017-09-11

**Authors:** Ye Jin Lee, Sun Mi Choi, Yeon Joo Lee, Young-Jae Cho, Ho Il Yoon, Jae-Ho Lee, Choon-Taek Lee, Jong Sun Park

**Affiliations:** 1 Division of Pulmonary and Critical Care Medicine, Department of Internal Medicine, Seoul National University College of Medicine, Seoul National University Hospital, Seoul, Korea; 2 Division of Pulmonary and Critical Care Medicine, Department of Internal Medicine, Seoul National University College of Medicine, Seoul National University Bundang Hospital, Seongnam, Korea; National and Kapodistrian University of Athens, GREECE

## Abstract

**Background:**

Although depression and anxiety represent significant yet treatable comorbidities in patients with idiopathic pulmonary fibrosis (IPF), their impact on the clinical course and prognosis of IPF remain unclear.

**Purpose:**

We investigated the prevalence and clinical significance of depression and anxiety in patients with IPF.

**Methods:**

The present study included a prospective cohort comprising 112 Korean patients with IPF who had completed the Hospital Anxiety and Depression Scale (HADS) questionnaire.

**Results:**

Symptoms of depression and anxiety were present in 25.9% and 21.4% of patients with IPF, respectively (HADS scores ≥8). No significant differences in demographic data, age, sex, smoking status, Modified Medical Research Council Dyspnea Scale (MMRC) scores, pulmonary function tests, or Gender-Age-Physiology Index for IPF were observed between patients with depression or anxiety and those without. However, in patients with anxiety, St. George's Respiratory Questionnaire (SGRQ) scores were significantly higher than those of patients without anxiety (40.5 *versus* 23.5; p = 0.003). The survival rate and total number of hospital admissions did not significantly differ between patients with depression/anxiety and those without.

**Conclusions:**

Our findings indicate that depression and anxiety are relatively common in patients with IPF. Although no significant differences were noted with regard to survival rate and hospitalization, the present study suggests that depression and anxiety significantly influence quality of life in patients with IPF.

## Introduction

Idiopathic pulmonary fibrosis (IPF) is a chronic, progressive interstitial lung disease of unknown cause that occurs primarily in older adults, with a median survival time of 2.5–3.5 years [1]. No curative medical treatment is available for IPF, and lung transplantation remains the only effective treatment. Therefore, patients with IPF may be prone to experience symptoms of depression and anxiety. Considering the high morbidity and mortality associated with IPF, improving the symptoms and quality of life (QOL) in such patients remains a priority in IPF research.

Several studies have reported that symptoms of depression and anxiety are common in patients with IPF. Such studies have indicated that the prevalence of depression ranges from 24.3–49.2%, while that of anxiety may be as high as 60%, in patients with IPF [2–4]. Additional studies have reported that depression and anxiety are more common in patients with severe, progressive forms of IPF [5]. Depression has been associated with the severity of dyspnea, cough, and pulmonary dysfunction [6, 7] and regarded as a major determinant of health-related quality of life (HRQL) in patients with IPF [8]. Although anxiety is also likely associated with poorer health status in patients with IPF, comparatively less is known regarding the association between anxiety and IPF.

The importance of depression and anxiety in chronic obstructive pulmonary disease (COPD) is well-known. Depression and anxiety are common in patients with COPD [9–11] and are known to negatively impact the course of the disease by increasing the severity of dyspnea, decreasing exercise capacity, and increasing physical disability and mortality [10, 12]. Previous studies have indicated that these associations may be due to decreased adherence to the treatment regimen and/or direct maladaptive physiologic effects [13, 14].

However, little is known regarding whether depression and anxiety affect clinical outcomes in patients with IPF. Furthermore, no studies to date have investigated the impact of treatment for depression/anxiety on QOL and pulmonary function in patients with IPF [15]. Therefore, in the present study, we investigated the impact of depression and anxiety on hospitalization and mortality associated with IPF using data from a prospective IPF registry.

## Materials and methods

### Study population and design

Patients were recruited for the present prospective study from among those in the Interstitial Lung Disease (ILD) Cohort of Seoul National University Bundang Hospital and Seoul National University Hospital between January 2012 and March 2015. All patients provided written informed consent based on institutional guidelines. The present study was approved by the Institutional Review Board and Ethics Committee of Seoul National University Bundang Hospital and Seoul National University Hospital (IRB no: B-1104/125-015, H-1304-018-477). Patients with IPF who had completed the Hospital Anxiety and Depression Scale (HADS) questionnaire were enrolled.

IPF was diagnosed after multidisciplinary discussions in accordance with criteria outlined by the 2011 consensus of the American Thoracic Society (ATS), the European Respiratory Society (ERS), the Japanese Respiratory Society, and the Latin American Thoracic Association [1]. The following characteristics were assessed during baseline clinical examinations: age, sex, smoking status, occupation, respiratory symptoms, comorbidities, pulmonary function test results (forced vital capacity [FVC], forced expiratory volume in 1 second [FEV1], and diffusing capacity of the lungs for carbon monoxide [DLCO]), HRQL, and depression/anxiety levels. Pulmonary function tests were performed by trained professionals in accordance with standardized ATS guidelines [16]. The Gender-Age-Physiology Index for IPF (GAP Index) was also calculated for each patient in accordance with methods described by Ley et al. [17].

Levels of depression and anxiety were assessed at baseline using the Hospital Anxiety and Depression Scale (HADS) [18]. In the present study, we utilized the validated Korean version of the HADS [19]. The HADS consists of seven items for depression (HADS-D) and seven items for anxiety (HADS-A), with scores on each subscale ranging from 0 to 21 points. Clinically significant depression and anxiety are indicated by scores ≥8 on both the HADS-D and HADS-A, respectively [20]. Thus, the prevalence of depression and anxiety in patients with IPF was calculated by assessing the percentage of participants with HADS scores ≥8.

HRQL was assessed using the validated St. George’s Respiratory Questionnaire (SGRQ). The SGRQ contains three subscales (symptom, activity, and impact). Total scores range from 0 to 100 [21], with higher scores indicative of poorer health status [22]. Although this questionnaire was originally developed for patients with obstructive lung disease, previous studies have validated its use in patients with IPF [23]. Dyspnea was assessed using the 5-grade Modified Medical Research Council (MMRC) Dyspnea Scale [24]. Numbers of hospitalizations for respiratory-related conditions such as pneumonia, aggravation of dyspnea, and acute exacerbation of IPF were also assessed. In cases of follow-up loss, survival status was obtained from the Ministry of Public Administration and Security.

### Statistical analysis

Chi-square (χ^2^) tests were used to compare categorical variables, whereas independent Student’s *t*-tests were used to compare continuous variables. Mortality was plotted in survival curves using Kaplan-Meier estimates and analyzed as the dependent variable using the Cox proportional hazards regression model. The multivariate Poisson regression model was also used to estimate the adjusted effects of depression and anxiety on the cumulative number of hospitalizations. All statistical analyses were performed using SPSS 21.0 (SPSS Inc., Chicago, IL, USA) and STATA 13 (STATA Corp, College Station, TX, USA). The level of statistical significance was set at P = 0.05.

## Results

### Baseline patient characteristics

**Fig 1:**
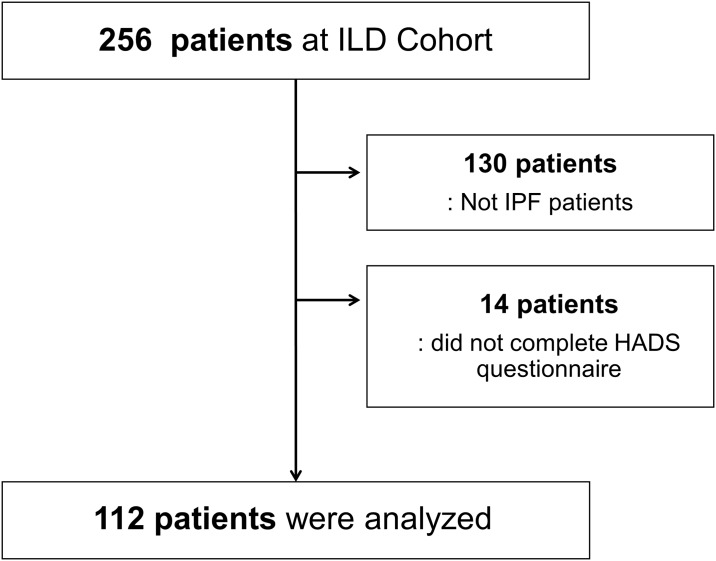
Study flow chart.

A total of 256 patients from the ILD Cohort were evaluated, 130 of whom were excluded due to diagnoses other than IPF. Fourteen patients were excluded because they did not complete the HADS questionnaire. A total of 112 patients with IPF were enrolled in the present study (Fig 1).

The baseline characteristics of the enrolled patients are presented in Table 1. Median patient age was 72 years (age range: 52–84 years, 75% male). The median follow-up duration was 23.6 months (range: 1–45.5 months).

**Table 1:** Baseline characteristics of the study patients.

Characteristic	All patients [N = 112]
**Median age [range] (years)**	72 [52–84]
**Male**	84 [75]
**FVC% pred**	85.0 ± 17.5
**DLCO% pred**	70.1 ± 23.1
**MMRC Dyspnea Score**	1.08 ± 0.96
**SGRQ**	
Impact	15.38 ± 17.30
Activity	42.84 ± 27.06
Symptom	36.82 ± 17.76
Total	27.11 ± 17.22
**Smoking status**	
Current smoker	11 [9.8]
Former smoker	67 [59.9]
Never smoker	34 [30.3]
**Comorbidity**	
DM	31 [27.7]
Hypertension	26 [23.2]
Coronary artery disease	24 [25]
COPD	9 [8]
Other lung disease*	29 [25.9]
**HADS score**	
HADS-A	3.46 ± 4.55
HADS-D	4.84 ± 4.17
**GAP score**	3.32 ± 0.15

Data are presented as n, median [interquartile], n [%], or mean ± SD. FVC: forced vital capacity; DLCO: diffusing capacity of the lungs for carbon monoxide; MMRC: Modified Medical Research Council; SGRQ: St. George’s Respiratory Questionnaire; DM: diabetes mellitus; COPD: chronic obstructive pulmonary disease; GAP: Gender-Age-Physiology; HADS-A: Hospital Anxiety and Depression Scale-Anxiety; HADS-D: Hospital Anxiety and Depression Scale-Depression.

*Other lung diseases included bronchiectasis, tuberculosis-destroyed lung, and asthma.

### Prevalence of depression and anxiety in patients with IPF

**Fig 2:**
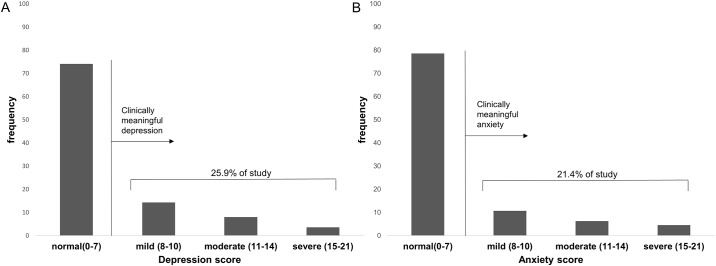
Prevalence of depression and anxiety in patients with IPF. (A) Prevalence of depression in patients with IPF. (B) Prevalence of anxiety in patients with IPF.

**Fig 3:**
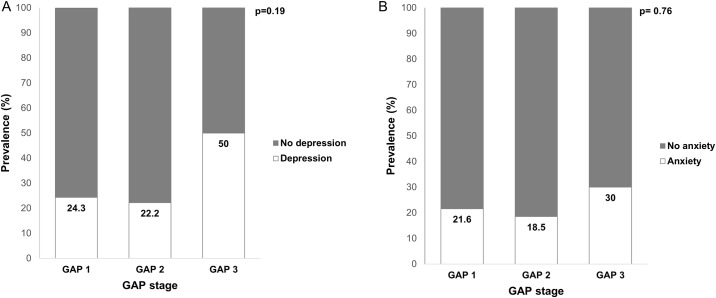
Prevalence of depression and anxiety according to GAP stage. (A) Prevalence of depression according to GAP stage. (B) Prevalence of anxiety according to GAP stage.

**Fig 4:**
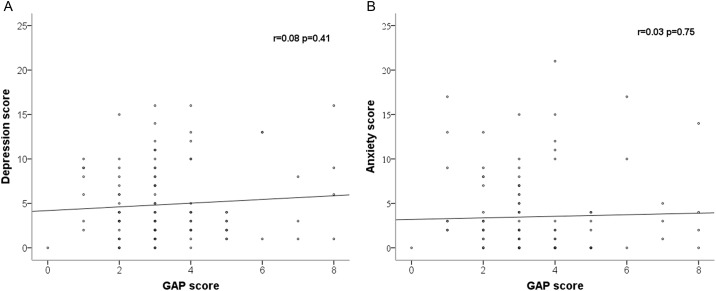
Associations between HADS scores and GAP score. (A) Association between HADS-A score and GAP score. (B) Association between HADS-D score and GAP score.

Among the 112 included patients, 29 patients (25.9%) exhibited symptoms of depression, while 24 patients (21.4%) exhibited symptoms of anxiety. The presence of depression and anxiety according to HADS scores are presented in Fig 2. No significant differences in the prevalence of depression or anxiety were observed according to GAP stage (Fig 3). Moreover, HADS scores were not correlated with GAP score (Fig 4).

### Comparisons between patients with or without depression and anxiety

No significant differences in age, sex, smoking status, number of comorbid conditions, MMRC Dyspnea Scale scores, and GAP Index values (score and stage) were observed between patients with and without depression/anxiety. However, patients with depression exhibited higher SGRQ Impact scores than patients without depression (23.4 vs 12.8, P = 0.036). Patients with anxiety exhibited higher total SGRQ (40.5 vs 23.5, P = 0.003) scores, Activity scores (56.5 vs 39.3, P = 0.021), and Impact scores (34.3 vs 10.4, P<0.001) than patients without anxiety. These results were similar between patients with symptoms of both depression and anxiety (14.3% of all patients) and those with neither depression nor anxiety (Table 2).

**Table 2:** Baseline characteristics according to presence of depression or anxiety.

	Depression	No depression	*P*	Anxiety	No anxiety	*P*	Both depression and anxiety	Neither depression nor anxiety	*P*
	(N = 29)	(N = 83)		(N = 24)	(N = 88)		(N = 16)	(N = 75)	
**Age**	71.76±7.43	70.57±7.52	0.46	68.79±9.19	71.44±6.90	0.20	70.9± 8.89	71.2±7.14	0.88
**Male sex**	22 (75.9%)	62 (74.7%)	0.90	19 (79.2%)	65 (73.9%)	0.79	14 (87.5%)	57 (76.0%)	
**Smoking**									
**Current**	4 (13.8%)	7 (8.4%)	0.54	4 (16.7%)	7 (8.0%)	0.48	4 (25.0%)	21 (28.0%)	0.43
**Former**	15 (51.7%)	52 (62.7%)		13 (54.2%)	54 (61.4%)		9 (56.3%)	48 (64.0%)	
**Never**	10 (34.5%)	24 (28.9%)		7 (29.2%)	27 (30.7%)		3 (18.8%)	6 (8.0%)	
**Smoking (PY)**	29.6±13.0	32.6±19.3	0.54	29.3±13.2	32.6±19.1	0.51	21.8±17.71	23.2±22.46	0.81
**SGRQ**									
**Symptom**	37.4±22.1	36.6±16.2	0.88	40.4±22.3	35.8±16.3	0.37	41.3±24.46	36.4±15.97	0.33
**Activity**	48.1±30.9	41.1±25.7	0.26	56.5±30.5	39.3±25.1	0.02	61.5±28.82	40.2±24.69	0.01
**Impact**	23.4±23.3	12.8±14.1	0.04	34.3±24.7	10.4±10.3	<0.001	31.8±25.9	9.92±9.12	<0.001
Total	32.2±22.8	25.4±14.8	0.16	40.5±24.0	23.5±12.9	0.003	39.8±24.80	23.7±12.37	<0.001
**Comorbidity**	1.0±0.9	1.1±1.0	0.86	1.0±1.1	1.1±0.9	0.74	1.25±1.06	1.12±0.99	0.64
**MMRC Dyspnea Score**	1.1±1.1	1.1±0.9	0.89	1.3±0.9	1.0±0.9	0.33	1.31±1.08	1.07±0.92	0.35
**FVC % predicted**	87.3±20.2	84.7±16.6	0.52	88.7±20.2	84.4±16.7	0.29	78.9±29.11	83.8±16.17	0.41
**DLCO % predicted**	73.9±29.4	69.9±20.9	0.35	71.4±29.2	69.8±21.4	0.81	71.1±36.53	67.7±23.46	0.64
**GAP score**	3.6±1.9	3.2±1.5	0.30	3.1 ± 1.8	3.4± 1.6	0.49	3.63±1.86	3.35±1.48	0.52
**GAP stage**	1.6±0.8	1.4±0.6	0.28	1.5±0.7	1.4±0.6	0.77	1.89±0.79	1.42±0.62	0.14

PY: pack years; FVC: forced vital capacity; DLCO: diffusing capacity of the lung for carbon monoxide; MMRC: Modified Medical Research Council; SGRQ: St. George’s Respiratory Questionnaire; GAP: Gender-Age-Physiology.

#### Mortality and hospital admission rate according to depression and anxiety status

**Fig 5:**
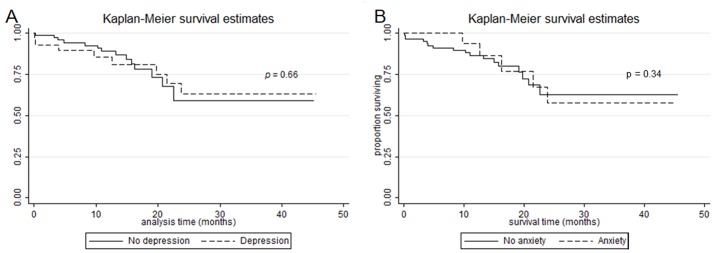
Kaplan-Meier plot of survival probability from the time of the initial visit stratified by depression and anxiety. (A) Mortality according to depression status. (B) Mortality of according to anxiety status.

The overall mortality rate in our study cohort was 12.5% (n = 14). Survival rate did not significantly differ according to depression and anxiety status (Fig 5). The hazard ratios of mortality for patients with depression and anxiety were 1.95 (95% CI, 0.63–6.03, P = 0.242) and 0.42 (95% CI, 0.10–1.80, P = 0.249), respectively, after adjusting for GAP scores, smoking status, and SGRQ score. Total numbers of hospital admissions did not significantly differ between patients with and without depression/anxiety (Table 3).

**Table 3:** Effect of depression and anxiety on hospital admission rate.

	No. of Patients	Adjusted Incidence Rate Ratio* (*95% CI*)
		Hospitalization
No depression (HADS-D≤7)	83	1 (reference)
Depression (HADS-D≥8)	29	1.07 (0.45–2.50)
p value for trend†		0.872
No anxiety (HADS-A≤7)	88	1 (reference)
Anxiety (HADS-A≥8)	24	1.55 (0.66–3.64)
p value for trend†		0.312

CI = Confidence interval; HADS = Hospital Anxiety and Depression Scale; HADS-A = HADS-Anxiety; HADS-D = HADS-Depression

*Adjusted for GAP score (age, sex, pulmonary function; forced vital capacity [FVC], diffusing capacity of the lungs for carbon monoxide [DLCO])

^†^ p value of testing for linear trend

## Discussion

In the present study, the prevalence of depression in patients with IPF was 25.9%, in accordance with the findings of studies conducted in Western countries, which have reported similar rates of depression among patients with IPF. Considering that the prevalence of major depressive disorder in the general Korean population is between 3.5~4.4% [25], our results indicate that patients with IPF are at increased risk for experiencing depression. There was no study for the mechanism of the increased prevalence of depression including anxiety among patients with IPF. However, as previous studies have indicated that depression is associated with dyspnea in ILD [4, 7], patients with IPF may be prone to be depression due to irreversible, progressive dyspnea. In our study, patients with depression exhibited reduced QOL relative to those without depression. Therefore, management of depression may be critical in improving QOL in patients with IPF.

Ryerson et al. [4] investigated depression in patients with ILD, reporting that the severity of dyspnea correlated with depression scores. Furthermore, the authors suggested that treatment of depression in patients with ILD may improve not only mood but also dyspnea. However, in the present study, we observed no association between depression and dyspnea grade or disease severity. It is possible that this lack of association is due to the milder conditions and relatively better lung function of patients in the present study (FVC% predicted 85.0±17.5%, SGRQ 27.11±17.22) relative to those of the Ryerson et al. study (74.3**±**18.5%).

In patients with COPD, depression is significantly associated with a higher risk of exacerbations and hospitalizations [26, 27]. However, depression identified in patients with IPF was not associated with increased mortality or hospitalization in the present study. This is likely because, unlike for COPD, there is no effective treatment regimen for improving IPF symptoms. Therefore, poor compliance with prescribed medical treatment due to depression is unlikely to affect treatment outcomes. However, it is also possible that the short follow-up period of the present study was responsible for this lack of association between depressive symptoms and patient outcomes. The mean follow-up duration was 23.6 months, which is shorter than the expected median survival time for IPF. Additional studies utilizing longer follow-up periods may therefore provide different results.

Several studies evaluating anxiety in IPF have demonstrated that the prevalence of anxiety is approximately 31–60% in patients with IPF and other ILDs [5, 7, 28]. In our study, the prevalence of anxiety in patients with IPF was 21.4%, although this discrepancy may be due to differences in measurement methods, region, and inclusion criteria. When patients are faced with irreversible and terminal conditions, anxiety tends to become more severe [29]. However, in the present study, we observed no association between anxiety and disease or dyspnea severity, consistent with the findings of a recent study involving a small sample of patients with ILD [7]. Although anxiety also influenced QOL in the present study (SGRQ, anxiety: 40.5±24.0, no anxiety: 23.5±12.9 p = 0.003), we observed no significant influence of anxiety symptoms on mortality or hospitalization.

To our knowledge, the present study is the first study to prospectively investigate the effects of depression and anxiety on clinical outcomes such as mortality and hospitalization in patients with IPF. Although neither depression nor anxiety exerted a significant influence on mortality or hospital admission rates, both depression and anxiety significantly influenced patient QOL. Unfortunately, we did not obtain data regarding psychological or pharmacological treatment with antidepressants or anxiolytics, nor did we attempt to manage depression or anxiety as assessed using the HADS. Therefore, we were unable to evaluate whether management of these symptoms leads to improved QOL. However, several recent studies have demonstrated that pulmonary rehabilitation programs involving educational sessions, disease management strategies, and exercise significantly improve functional capacity, dyspnea, and QOL in patients with IPF [30, 31]. As depression and anxiety were highly prevalent and influenced QOL in patients of the present study, the accumulated evidence indicates that patients with IPF are likely to benefit from cognitive behavioral therapy and counseling by qualified healthcare professionals. Nonetheless, further studies are required to determine whether psychological and pharmacological treatment for depression and or anxiety can improve health outcomes such as QOL, mortality, hospitalization, and exacerbation of symptoms in patients with IPF.

## Conclusions

In conclusion, our findings indicate that depression and anxiety are common in Asian patients with IPF. Although neither depression nor anxiety was associated with mortality or hospital admission rate, the presence of depression or anxiety significantly influenced patient QOL, highlighting the need for improved detection and treatment of such conditions in this patient population. Future prospective studies are required to investigate the effect of antidepressants and anxiolytics on QOL and determine the prognostic significance of depression and anxiety in patients with IPF.

## Supporting information

S1 Data Set Raw data_PLOSONE(ID delete).final. (XLS)

## Abbreviations

ATS: American thoracic society
COPD: chronic obstructive pulmonary disease
DLCO: diffusing capacity of the lungs for carbon monoxide
ERS: European Respiratory Society
FEV1: forced expiratory volume in 1 second
FVC: forced vital capacity
GAP: gender-age-physiology
HADS: Hospital Anxiety and Depression Scale
ILD: interstitial lung disease
IPF: Idiopathic pulmonary fibrosis
MMRC: Modified Medical Research Council
QOL: quality of life
SGRQ: St. George's Respiratory Questionnaire
